# Nutritional Deficiencies and Associated Oral Health in Adolescents: A Comprehensive Scoping Review

**DOI:** 10.3390/children11070869

**Published:** 2024-07-18

**Authors:** Man Hung, Amy Blazejewski, Samantha Lee, Johanna Lu, Andres Soto, Connor Schwartz, Amir Mohajeri

**Affiliations:** 1 College of Dental Medicine, Roseman University of Health Sciences, South Jordan, UT 84095, USA; 2 Department of Family and Preventive Medicine, University of Utah, Salt Lake City, UT 84108, USA; 3 Department of Orthopaedic Surgery Operations, University of Utah, Salt Lake City, UT 84108, USA; 4 Department of Educational Psychology, University of Utah, Salt Lake City, UT 84109, USA; 5 The Wharton School, University of Pennsylvania, Philadelphia, PA 19104, USA; 6 Huntsman Cancer Institute, Salt Lake City, UT 84112, USA; 7 Library, Roseman University of Health Sciences, South Jordan, UT 84095, USA

**Keywords:** nutrition, children, adolescent, health, dentistry, diet

## Abstract

Introduction: The shift to processed foods in American diets has increased vitamin and mineral deficiencies among adolescents, impacting growth and health, often manifesting as oral lesions. This review study aimed to explore the link between nutritional deficiencies and adolescent oral health to improve guidance and prevent long-term issues. Methods: A systematic review of literature from 2013 to 2023 was conducted on adolescents aged 10–19 years, using PRISMA guidelines. Searches in PubMed, Web of Science, Dentistry & Oral Sciences Source—Ebscohost, and Scopus included peer-reviewed articles, excluding reviews and non-empirical studies. Data were screened and extracted with independent reviews for accuracy. Results: Malnutrition strongly correlates with poor oral health. Undernourished children have a 60% increase in dental caries, exacerbated by high sugar intake. Early malnutrition delays dental eruption, temporarily protecting against caries, while stunting and infections cause enamel defects. Chronic conditions like cerebral palsy and celiac disease worsen oral health, with risks persisting into adulthood. Conclusions: Nutritional deficiencies and oral health are interconnected, requiring integrated healthcare. Early interventions and holistic strategies can improve outcomes and reduce long-term burdens. Comprehensive health education and routine dental evaluations are essential for prevention and treatment, enhancing health across all demographics.

## 1. Introduction

Vitamin and mineral deficiencies represent a prevalent and often unrecognized problem among adolescents. The ever-changing model of food production in America, compounded by inflation, poverty, and the rise of fad diets, has led to an increase in the consumption of processed foods [1]. Consequently, there has been a reduction in the consumption of whole fruits, vegetables, and quality meats. These changes in dietary habits have significantly impacted the overall nutritional status of individuals, especially adolescents.

As disorders such as Crohn’s disease or deficiencies in vitamins and minerals progress, observable signs and symptoms emerge. Deficiencies can manifest in various ways, affecting energy levels or causing hair loss, prompting affected individuals to seek treatment [2]. Conversely, some deficiencies, such as a deficiency in calcium, may develop gradually, going unnoticed and undiagnosed until identified by a trained clinician during examination. Importantly, certain deficiencies manifest in the oral cavity, providing critical clinical clues about underlying nutritional issues [3]. The oral cavity is often one of the first sites where nutritional deficiencies become apparent. Therefore, it is essential for clinicians to receive appropriate training to correctly identify how different deficiencies and undiagnosed disorders manifest in the oral cavity, enabling early diagnosis. Despite the significance of this issue, there is limited research on oral lesions associated with nutritional deficiencies in adolescents. For instance, Pflipsen et al., in a paper published by the Academy of General Dentistry, explain how vitamin D deficiencies can lead to dentin and enamel hypoplasia [4]. They highlight that infants who are exclusively breastfed and older individuals are particularly susceptible to vitamin D deficiency. However, their study does not address vitamin D intake levels among adolescents or the prevalence of deficiency risk within this age group. Oral lesions can also be related to other vitamin deficiencies. For example, vitamin B2 (riboflavin) is essential for energy production and cellular function, and its deficiency can cause glossitis and angular stomatitis [5]. Vitamin B3 (niacin) is important for DNA repair and metabolism, with deficiencies leading to oral symptoms like stomatitis, glossitis, and mucosal ulceration [6]. Vitamin B9 (folate) is necessary for DNA synthesis and repair, and cell division, and its deficiency can lead to recurrent aphthous stomatitis and oral ulcerations [7]. Vitamin C (ascorbic acid) is important for collagen synthesis, antioxidant function, and immune support; its deficiency causes scurvy, leading to damage of periodontal ligaments and oral microbiome alterations [8]. Vitamin E functions as an antioxidant, protecting cell membranes from oxidative damage, and its deficiency can contribute to various oral health issues [9]. Together, these vitamins are integral to preventing and managing oral lesions. Thus, it is crucial to identify which individuals are at highest risk of developing caries and how these risks can be mitigated.

Having a healthy body starts by having a balanced nutritious diet. Having sufficient intake of essential nutrients and vitamins is critical to also having a healthy mouth. However, in recent years, increased access and promotion of processed and sugary foods have been an appeal to many, especially among adolescents. This should be alarming as studies show that malnutrition during childhood can negatively affect proper tooth formation and immunological functioning by decreasing salivary flow, which in turn increases adolescents’ susceptibility to dental caries [10]. Nutrition has significant effects in the oral cavity not only on the development of dental decay and enamel erosion, which are related to dietary acids and sugars seen in foods like soft drinks, but also influences on craniofacial development, oral cancer, and other oral infectious diseases [11]. Therefore, early and preventative measures such as interventions focusing on incorporating healthy eating habits among this population are vital for their wellbeing. 

Diet counseling with a child’s parents or caregivers during a dental appointment is an integral part of anticipatory guidance. At a young age, children are not primarily responsible for making their food choices. Once children reach adolescence, the decision-making role shifts to them, rather than their parents, when it comes to food choices. Naidoo et al. [12] describe specific guidelines that parents can follow to mitigate caries risk in their children, such as not sending children to bed with a bottle and restricting cow’s milk within the first year of life [13]. Further investigation is needed to identify common eating habits during adolescence that reflect nutritional deficiencies and cause oral lesions, to promote healthier habits and oral health.

Inadequate intake of essential vitamins and minerals affects diurnal bodily functions and is critical for optimal growth, especially during adolescence. Poor nutritional intake can have permanent impacts, such as stunted growth, weakened immune systems, digestive issues, poor wound healing, and poor bone development. This scoping review sought to examine the common patterns and themes in which these deficiencies and disorders manifest, to better serve patients and ensure they receive appropriate treatment. Additionally, the review aimed to identify the most common deficiencies in adolescents and their impact on growth patterns. Having the appropriate knowledge and preparedness as clinicians allows for the adoption of a preventive philosophy and the ability to make informed food choice recommendations or referrals to dietitians before problems permanently affect patients’ overall health or growth outcomes.

## 2. Methods

### 2.1. Article Eligibility Criteria

The Preferred Reporting Items for Systematic Reviews and Meta-Analyses (PRISMA) Guidelines were utilized as a methodological framework to ensure a rigorous and standardized approach in evaluating the extant literature. Research articles were deemed eligible for inclusion if they specifically addressed nutritional deficiencies pertaining to oral health in adolescents, as defined by the World Health Organization for individuals aged 10 to 19 years [14]. In addition, eligible articles had to undergo peer review and be published in English between 2013 and 2023, while exclusion criteria targeted abstract-only presentations, articles lacking full text, review articles, and articles over 10 years old.

To maintain a focus on original research, review articles providing summaries of existing literature were intentionally omitted. Moreover, conference proceedings, opinion pieces, and letters to the editor were excluded to uphold strict criteria aimed at including empirical evidence. A comprehensive outline of the inclusion and exclusion criteria guiding the article selection process is detailed in Table 1.

### 2.2. Database Sources and Search Strategies

The initial search was conducted across multiple databases, namely PubMed, Web of Science, Dentistry & Oral Sciences Source—Ebscohost, and Scopus. These databases were selected due to their extensive coverage of scholarly articles, clinical trials, and research studies across diverse fields. This deliberate selection aimed to broaden the scope of sources included in the review, encompassing a wide array of scientific contributions.

Specific search strategies and keywords tailored to each database were developed and implemented. A detailed outline of these strategies is provided in Table 2. The formulation of these strategies was characterized by careful attention to detail, ensuring their customized nature to optimize the retrieval of relevant articles. This methodological approach was crafted to facilitate a comprehensive and exhaustive examination of relevant studies within the realm of nutritional deficiencies concerning oral health in adolescents.

### 2.3. Data Collection

During the initial screening phase, a team of four authors (A.B., S.L., A.S., and J.L.) was tasked with examining the titles and abstracts of articles to ascertain their suitability. This involved ensuring that the articles were peer-reviewed, involved human subjects, were written in English, and were relevant to the topic of interest. In cases where it was unclear whether articles matched the implemented search strategies, other authors, C.S. and A.M., conducted a secondary screening of the articles in question. 

To extract data, a pre-established form in Excel (Version 2024) was employed to systematically condense details regarding study design, participant demographics, and pertinent findings. Each article was scrutinized by two independent reviewers. Initially, A.B., S.L., A.S., and J.L. each independently assessed the articles. After this initial review, these same authors conducted a secondary review of each other’s work to reconcile any disparities and ensure consistency. Additionally, a third reviewer, A.M., cross-checked the data to verify its accuracy and comprehensiveness. Once all reviews were completed, M.H. conducted an additional review focusing on the quality of the studies, the sources of the data, and the thoroughness of the data charting. This final review further enhanced the robustness and reliability of the scoping review process.

## 3. Results

**Figure 1:**
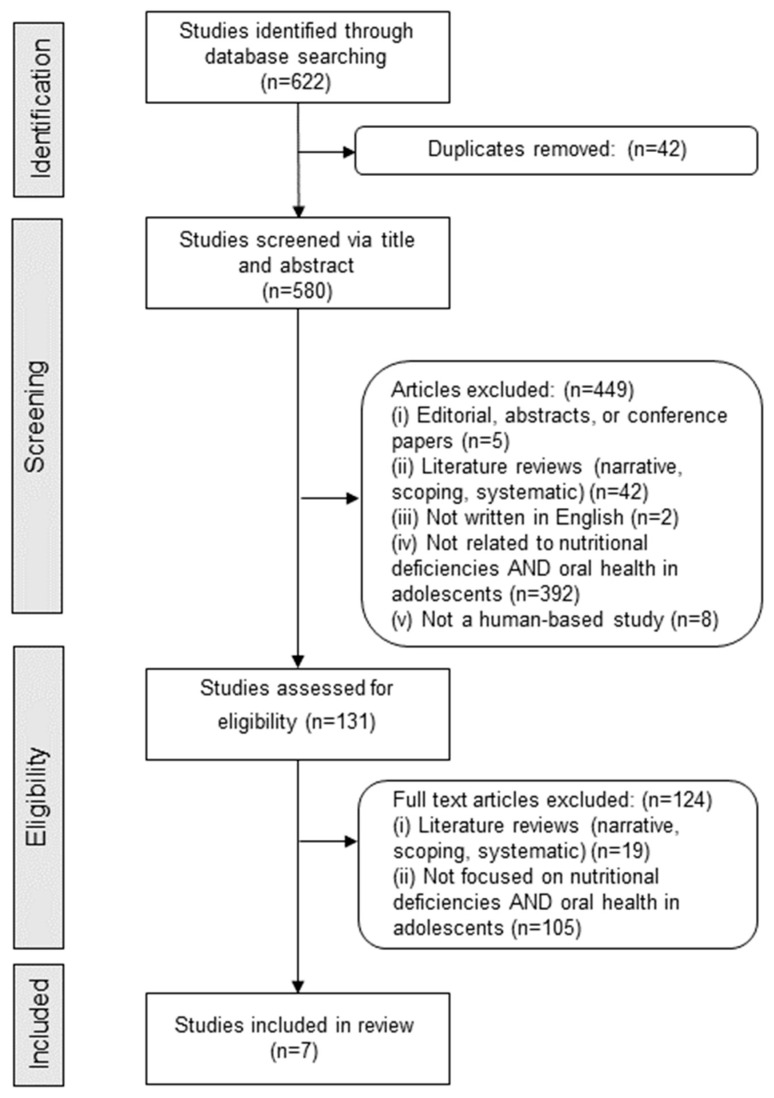
Flow diagram of article selection.

The initial search across multiple databases yielded a total of 622 articles. After the removal of 42 duplicates, 580 articles remained for the screening process. The titles and abstracts of these 580 articles were reviewed, leading to the exclusion of 449 articles for various reasons: five were editorials, abstracts, or conference papers; 42 were literature reviews; two were not written in English; 392 were not related to nutritional deficiencies and oral health in adolescents; and 8 were not human-based studies. This initial screening resulted in 131 articles being assessed for eligibility through full-text review. Upon further evaluation, 124 articles were excluded, with 19 identified as literature reviews and 105 not focused on the specific intersection of nutritional deficiencies and oral health in adolescents. Consequently, seven studies met all the inclusion criteria and were selected for the final review (Figure 1).

The data from various studies highlight important findings related to malnutrition, oral health, and specific medical conditions (Table 3). The results are categorized into three overarching themes: Link Between Nutritional Deficiencies and Oral Health, Chronic Conditions and Their Effects on Oral Health, and Oral Health as a Reflection of General Health.

### 3.1. Link between Nutritional Deficiencies and Oral Health

The data illustrate a pronounced relationship between nutritional deficiencies and detrimental outcomes in oral health. Rego et al.’s study highlights that undernutrition significantly raises the prevalence of dental caries, with underweight children experiencing a 60% increase in decayed teeth compared to their counterparts with normal nutritional status [10]. This effect is exacerbated by high sugar consumption. Reyes et al.’s findings complement this by demonstrating an inverse relationship between early childhood protein–energy malnutrition and the presence of decayed, missing, and filled teeth among Haitian children [16], suggesting that malnutrition-related delays in dental eruption may offer a temporary shield against caries. Masterson’s research supports these findings by linking stunting and parasitic helminth infections in early childhood to visible enamel defects [17], underscoring the impact of nutritional status during critical developmental periods on later dental health.

### 3.2. Chronic Conditions and Their Effects on Oral Health

Research by Costa et al. shows that students with cerebral palsy frequently suffer from a cluster of health issues including oropharyngeal dysphagia, malnutrition, dehydration, and compromised oral health [19], pointing to a multifaceted relationship between chronic disorders and oral health maintenance. In parallel, Cruz et al.’s study indicates that individuals with celiac disease are notably more susceptible to dental enamel defects and a dry mouth sensation [18], highlighting how systemic diseases can directly influence oral health outcomes.

### 3.3. Oral Health as a Reflection of General Health

Fitri et al.’s analysis provides insight into the oral health status among stunted children, revealing that 71% of these individuals had poor Oral Hygiene Index-Simplified (OHI-S) scores, with only 1% showing good scores [20]. This prevalence of poor oral hygiene, particularly among male children in the study, suggests a direct correlation between stunting and diminished oral health. Heinrich et al.’s findings further suggest that the common risk factors for impaired growth and dental development not only manifest in childhood but also extend into adulthood, emphasizing the enduring impact of these early-life challenges on long-term health [15].

## 4. Discussion

This study delves into the intricate connections between nutrition, chronic conditions, and oral health, uncovering three themes that encapsulate the core findings. The first theme explores the interplay between nutritional deficiencies and oral health, emphasizing the critical impact of undernutrition and high sugar intake on dental caries and other oral health issues. The second theme examines the complex relationship between chronic conditions and oral health, highlighting how various chronic diseases exacerbate oral health problems through multifaceted mechanisms. The third theme reflects on how oral health serves as an indicator of general health, particularly in the context of childhood nutritional status and its long-term implications.

### 4.1. Interplay between Nutritional Deficiencies and Oral Health

The relationship between nutritional deficiencies and adverse oral health outcomes is strongly supported by the literature. Rego et al. highlight a significant increase in dental caries among undernourished children, noting that undernutrition is a critical risk factor for dental decay exacerbated by high sugar intake [10]. This aligns with findings from other studies, including a study done in the United Kingdom by Hong et al., which emphasizes that excessive sugar consumption, coupled with inadequate nutritional status, significantly raises the risk of dental caries [21]. Moreover, evidence from similar research points to the fact that high sugar diets impair the body’s ability to utilize essential nutrients, thereby compounding oral health issues [22].

Reyes et al. introduce an interesting dynamic where malnutrition-related delays in dental eruption might temporarily protect against caries [16]. Their findings suggest that delayed dental eruption due to malnutrition could reduce the exposure time of teeth to cariogenic challenges, thus offering temporary protection against dental caries. This phenomenon is supported by other studies, such as the one performed by Heinrich-Weltzien et al. in 2013, which show that malnutrition can affect the timing of tooth eruption, which may have short-term protective effects [23].

Furthermore, Masterson’s study on the connection between early childhood malnutrition and enamel defects emphasizes the long-lasting impact of early-life nutritional deficits [17]. Enamel defects, such as hypoplasia, are prevalent in populations with high rates of malnutrition and are associated with increased susceptibility to dental caries [24]. Clinically, oral health care providers should look for pitting in the enamel of teeth that is accompanied by brown, caramel-colored discoloration. These enamel defects can present themselves on every surface of the teeth, such as the facial, lingual, buccal, and occlusal surfaces, and can affect any tooth in the mouth. These findings underscore the need for healthcare strategies that address both immediate dietary needs and long-term oral health. An effective strategy that clinicians can use to understand a patient’s eating habits is to utilize a 24 h dietary recall, which includes questions for the child or caregiver that prompt the open-ended response of the patient. These may include, for example, asking what they had for breakfast in the past 24 h, following up with detailed questions about what they had to drink, if they added cream or sugar in their coffee, or about what sort of spread (butter, jam) they used on top of a snack or toast to encourage memory recall of their habits. The use of food models, pictures, and visual aids that represent portion sizes can help respondents report food intake and portions more accurately [25]. For example, the palm of an adult hand can represent 3 oz equivalents, whereas a fist can represent one cup. The clinician can then recommend substitutes that are reasonably achievable, such as artificial sweeteners in coffee or fresh fruit on top of toast instead of a spread to include fiber and essential vitamins. Alternatively, the clinician should know of at least one reputable dietitian in their network that they can refer patients to in order to tackle nutritional education in a comprehensive way with the patient and/or caregiver.

### 4.2. Chronic Conditions Impacting Oral Health

The relationship between chronic conditions and oral health is complex and multifaceted. Costa et al. demonstrate that individuals with cerebral palsy frequently experience numerous health issues including oropharyngeal dysphagia, malnutrition, and dehydration, which collectively impair oral health [19]. This is consistent with other research showing that neurological disorders often lead to significant oral health problems due to factors such as impaired motor function and difficulty maintaining oral hygiene [26].

Similarly, Cruz et al. identify celiac disease as a significant factor increasing the risk of dental enamel defects and dry mouth [18]. Studies have shown that the malabsorption and immune responses associated with celiac disease can lead to dental issues such as enamel hypoplasia and recurrent aphthous stomatitis [27]. Research has also highlighted the association between amelogenesis imperfecta and its manifestations of enamel hypoplasia, enamel hypocalcification, and hypo-maturation, and its connection to systemic conditions that can coexist, like nephrocalcinosis [28]. It is important that dentists are knowledgeable of this association to aid in the early detection of renal abnormalities through these clinical presentations to get patients the help they need earlier. Amelogenesis imperfecta can present itself as enamel that is softer and duller, often opalescent, and can appear opaque-white or honey-colored. These findings highlight the necessity for specialized dental care protocols that address the unique challenges faced by individuals with chronic conditions.

### 4.3. Oral Health Reflecting General Health

Fitri et al.’s analysis of the OHI-S scores among stunted children reveals a disturbing trend of poor oral hygiene, especially among male children [20]. This mirrors findings from other studies that link poor nutritional status to a range of oral health issues, indicating that stunting and poor oral hygiene are interrelated problems that reflect broader systemic health deficiencies [11].

Children affected by chronic kidney disease (CKD) require improved health interventions. One common issue for these children is halitosis, caused by excess ammonia being exhaled through the lungs. Hoefer et al. found that CKD patients with halitosis who underwent dental prophylaxis every three months had lower amounts of volatile sulfur compounds compared to those who had prophylaxis every six months [29]. Implementing more frequent dental maintenance appointments for prophylaxis can be a beneficial modification for reducing halitosis in these highly vulnerable patients.

Heinrich et al. suggest that risk factors for impaired growth and dental development observed in childhood can persist into adulthood, indicating long-term health consequences [15]. This underscores the importance of early nutritional interventions to mitigate lifelong dental and general health issues. Similar conclusions are drawn by studies showing that early-life health interventions can have a profound impact on long-term health outcomes, emphasizing the need for integrated healthcare approaches [30].

### 4.4. Implications

The findings from these studies have several implications for public health policies and individual healthcare practices. The strong link between nutrition and oral health necessitates the integration of dental health considerations into nutritional programs. Health education should emphasize the importance of a balanced diet for both general and oral health. It can include instructing mothers to refrain from sending their children to bed with a bottle or sippy cup containing milk or juice [31]. It is important to educate that frequently sipping on a sugar sweetened beverage, even when the sugar is naturally found in milk, continuously lowers the pH of the oral cavity and increases the risk of caries development. Encouraging their children to consume milk or other sugar sweetened beverages at a minimum and to finish it during a specific time frame encourages that the teeth are not continuously exposed to sugar. It is important to reinforce that water is the best option for drinking during the evening time if the child becomes thirsty. Additionally, routine oral health assessments should be incorporated into standard medical evaluations, especially for patients with chronic conditions or nutritional deficiencies. This approach is supported by evidence suggesting that comprehensive health assessments can improve both general and oral health outcomes.

Developing specialized dental care protocols for individuals with chronic conditions is also imperative. These protocols should address the specific needs of these patients, ensuring comprehensive care that encompasses both general and oral health. Tailoring dental care to meet the unique challenges faced by these populations can significantly improve their quality of life and overall health. It is essential for health care providers, especially dentists, to have an understanding of chronic conditions such as cerebral palsy and celiac disease so that they can provide individualized care for patients that need extra modifications. For example, having the knowledge that cerebral palsy and celiac disease increase the risk of dry mouth and therefore the risk of dental caries, the dentist can place these patients on more frequent dental recall and prophylactic appointments of 3-month intervals. In doing so, they can better monitor high-risk patients and mitigate caries development so caries have less time to progress and develop between appointments. At these appointments, the dental provider can administer topical fluoride varnish applications at more frequent intervals to remineralize the dental enamel and reduce the risk of caries [32]. In addition, dentists can recommend sugar-free lozenges to these patients, after determining that they can safely tolerate lozenges without choking risk, to stimulate saliva production to balance the pH of the saliva and reduce caries risk. These are two examples of concrete modifications that dental providers can make to better serve specific populations with chronic diseases.

### 4.5. Strengths and Limitations

A significant strength of this study is its rigorous methodological approach, adhering to the PRISMA guidelines to ensure a comprehensive and systematic review of the literature. By including only peer-reviewed articles published in English from 2013 to 2023 and excluding reviews, abstract-only presentations, and non-empirical contributions, the study ensures the reliability and relevance of the data analyzed. The thorough search across multiple databases, combined with independent reviews and cross-checks of data, further enhances the accuracy and detail of the findings, providing robust evidence on the link between nutritional deficiencies and oral health in adolescents. This process underscores the study’s credibility and its potential to inform effective dietary interventions and public health strategies in dental settings. Additionally, this study provides critical insights on the connections between nutrition, chronic conditions, and oral health. It highlights the impact of nutritional deficiencies and high sugar intake on oral health, the exacerbation of oral health problems by chronic diseases, and the role of oral health as an indicator of general health in children.

Despite the critical insights provided by these studies, there are limitations that need to be addressed. Most studies are observational and primarily establish correlations rather than causations, limiting the ability to definitively conclude that nutritional deficiencies cause poor oral health. Other factors such as socioeconomic status, healthcare access, and genetic predispositions may also play a role. Additionally, the geographic and demographic diversity of study participants is often limited, affecting the generalizability of the findings across different populations. These limitations highlight the need for more rigorous research designs to better understand these relationships.

### 4.6. Future Research

Future research should aim to establish causative relationships between nutritional status and oral health outcomes through longitudinal studies. Clinical trials are needed to test the effectiveness of integrated health interventions that address both nutrition and oral health. Expanding the demographic and geographic diversity of research participants will be crucial to understanding the global applicability of the findings. Additionally, studies exploring the impact of specific nutritional interventions on oral health outcomes in populations with chronic diseases could provide targeted insights for healthcare practice.

## 5. Conclusions

The interconnectedness between nutritional deficiencies, chronic health conditions, and oral health is evident across diverse studies, highlighting significant implications for both healthcare policy and practice. These findings underscore the importance of holistic health approaches that integrate nutrition, dental care, and chronic disease management. Addressing these issues through early intervention and integrated care strategies can significantly enhance health outcomes and reduce long-term healthcare burdens: (1) Interventions that should be prioritized in the healthcare setting include education for both children and caregivers. This education should cover dietary recalls, diet modification, and the importance of limiting high-sugar drinks at bedtime. Additionally, educating clinicians is essential. Enhanced inter-professional collaboration among providers, such as dentists, pediatricians, and registered dietitians, is necessary to enable more comprehensive care management for patients. (2) By further exploring the causative links between diet, systemic health, and oral health outcomes, future research can pave the way for more effective public health interventions. (3) Implementing comprehensive health education and integrating dental evaluations into routine health assessments could significantly improve both prevention and treatment strategies, ultimately leading to better health across lifespans and demographics.

## Figures and Tables

**Table 1:** Inclusion/exclusion criteria for article selection.

Inclusion Criteria	Exclusion Criteria
Peer-reviewed research articles in English Participants aged 10 to 19 years Focused on nutritional deficiencies pertaining to oral health in adolescents Articles published between 2013 and 2023 Human-based studies	Articles that consisted of only abstracts without the full text Animal studies Literature reviews (narrative, scoping, systematic, meta-analysis) Conference proceedings Opinion articles (letter to editor, editorial comments, opinion)

**Table 2:** Database search strategies.

Databases (Date of Search)	Search Strategies	Number of Articles Found
Dentistry & Oral Sciences Source—Ebscohost (13 September 2023)	((“Malnutrition”) OR (“Nutritional Deficiency”) OR (“Nutritional Deficiencies”) OR (“Undernutrition”) OR (“Malnourishment”) OR (“Malnourishments”)) AND (((“Adolescent”) OR (“Adolescents”) OR (“Adolescence”) OR (“Teens”) OR (“Teen”) OR (“Teenagers”) OR (“Teenager”) OR (“Youth”) OR (“Youths”) OR (“Adolescents, Female”) OR (“Adolescent, Female”) OR (“Female Adolescent”) OR (“Female Adolescents”) OR (“Adolescent, Male”) OR (“Adolescents, Male”) OR (“Male Adolescent”) OR (“Male Adolescents”)) OR ((“Child”) OR (“Children”)) OR ((“Child, Preschool”) OR (“Preschool Child”) OR (“Children, Preschool”) OR (“Preschool Children”)) OR ((“Infant”) OR (“Infants”)) OR (“Growth Chart”)) AND ((“Mouth”) OR (“Oral Cavity”) OR (“Cavity, Oral”) OR (“Cavitas Oris”) OR (“Vestibule of the Mouth”) OR (“Oral Cavity Proper”) OR (“Mouth Cavity Proper”) OR (“Cavitas oris propria”) OR (“Oral Health”)) AND ((men) OR (women) OR (patient) OR (female) OR (male) OR (subjects) OR (adult) OR (human)) NOT (animal models) Filter: Human AND 2013–2023	15
PubMed (13 September 2023)	(“Malnutrition”[Mesh]) AND (“Adolescent”[Mesh] OR “Child”[Mesh] OR “Child, Preschool”[Mesh] OR “Infant”[Mesh] OR “growth chart”) AND (“Mouth”[Mesh] OR “oral cavity” OR “oral health”) Filter: Human AND 2013–2023	87
Scopus (13 September 2023)	(TITLE-ABS-KEY (((“Malnutrition”) OR (“Nutritional Deficiency”) OR (“Nutritional Deficiencies”) OR (“Undernutrition”) OR (“Malnourishment”) OR (“Malnourishments”))) AND TITLE-ABS-KEY ((((“Adolescent”) OR (“Adolescents”) OR (“Adolescence”) OR (“Teens”) OR (“Teen”) OR (“Teenagers”) OR (“Teenager”) OR (“Youth”) OR (“Youths”)) OR ((“Child”) OR (“Children”)) OR ((“Child, Preschool”) OR (“Preschool Child”) OR (“Children, Preschool”) OR (“Preschool Children”)) OR ((“Infant”) OR (“Infants”)) OR (“Growth Chart”)))) AND ((dental) OR (dentist)) AND NOT (“animal models”) AND (LIMIT-TO (PUBYEAR,2013) OR LIMIT-TO (PUBYEAR,2014) OR LIMIT-TO (PUBYEAR,2015) OR LIMIT-TO (PUBYEAR,2016) OR LIMIT-TO (PUBYEAR,2017) OR LIMIT-TO (PUBYEAR,2018) OR LIMIT-TO (PUBYEAR,2019) OR LIMIT-TO (PUBYEAR,2020) OR LIMIT-TO (PUBYEAR,2021) OR LIMIT-TO (PUBYEAR,2022) OR LIMIT-TO (PUBYEAR,2023)) AND (LIMIT-TO (LANGUAGE,”English”)) AND (LIMIT-TO (EXACTKEYWORD,”Human”) OR LIMIT-TO (EXACTKEYWORD,”Humans”) OR EXCLUDE (EXACTKEYWORD,”Review”)) Filter: Human AND 2013–2023 AND Exclude Reviews and English	515
Web of Science (13 September 2023)	((((TS = (((“Malnutrition”) OR (“Nutritional Deficiency”) OR (“Nutritional Deficiencies”) OR (“Undernutrition”) OR (“Malnourishment”) OR (“Malnourishments”)))) AND TS = ((((“Adolescent”) OR (“Adolescents”) OR (“Adolescence”) OR (“Teens”) OR (“Teen”) OR (“Teenagers”) OR (“Teenager”) OR (“Youth”) OR (“Youths”) OR (“Adolescents, Female”) OR (“Adolescent, Female”) OR (“Female Adolescent”) OR (“Female Adolescents”) OR (“Adolescent, Male”) OR (“Adolescents, Male”) OR (“Male Adolescent”) OR (“Male Adolescents”)) OR ((“Child”) OR (“Children”)) OR ((“Child, Preschool”) OR (“Preschool Child”) OR (“Children, Preschool”) OR (“Preschool Children”)) OR ((“Infant”) OR (“Infants”)) OR (“Growth Chart”)))) AND TS = (((“Mouth”) OR (“Oral Cavity”) OR (“Cavity, Oral”) OR (“Cavitas Oris”) OR (“Vestibule of the Mouth”) OR (“Oral Cavity Proper”) OR (“Mouth Cavity Proper”) OR (“Cavitas oris propria”) OR (“Oral Health”))))) NOT ALL = ((animal models)) Filter: Human AND 2013–2023 AND English	5

**Table 3:** Study summaries.

Author (Year)	Country	Sample Size	Age Range (Years)	Study Aims	Study Design	Outcomes
Heinrich-Weltzien et al., (2013) [15]	Philippines	1554	10 to 13 years	Observe any delay in the eruption of permanent teeth among Filipino adolescents with stunting or thinness	Cross-sectional	Impaired physical growth and dental development have common risk factors that persist into adulthood
Reyes-Perez et al., (2014) [16]	United States	1058	11 to 19 years	Determine the effect of early childhood protein–energy malnutrition on decayed, missing, and filled tooth scores in the permanent dentition of rural Haitian adolescents aged 11–19 years	Cohort study	Protein–energy malnutrition status is inversely associated with decayed, missing, and filled tooth scores in Haitian participants
Masterson et al., (2017) [17]	Bolivia	349	10 to 17	Investigate the relationship between early childhood malnutrition-related measures and subsequent enamel defects in the permanent dentition	Cohort study	Evidence for associations of malnutrition-related measures in early childhood, including stunted growth and parasitic helminth infection, with the observed enamel defects
Cruz et al., (2018) [18]	Brazil	80	Median age of 16.5	Evaluate the dental and oral manifestations in patients with celiac disease	Cross-sectional	Celiac disease increased the likelihood of dental enamel defects and dry mouth sensation; the oral examination can be an important auxiliary tool for the identification of cases of the disease
Rego et al., (2020) [10]	Brazil	406	12 years	Investigate the relationship between nutritional status and dental caries in 12-year-old low-income children	Cross-sectional	Underweight children had a 60% higher mean of decayed teeth than children with normal nutritional status; underweight children with high annual sugar intake had a greater mean of decayed teeth than underweight children with low sugar intake; malnutrition is associated with dental caries among children from low-income families
Costa et al., (2021) [19]	Spain	33	-	Assess the prevalence of oropharyngeal dysphagia, malnutrition, dehydration, and oral health in students at a special needs school	Cross-sectional	Malnutrition, dehydration, oropharyngeal dysphagia, and poor oral health are highly prevalent conditions in students with cerebral palsy
Fitri et al., (2023) [20]	Indonesia	76	10 to12 years	Describe the Oral Hygiene Index-Simplified scores in stunted children	Cross-sectional	The study showed that 71% of subjects had poor Oral Hygiene Index-Simplified scores, 28% had moderate scores, and only 1% of subjects had good scores; male children dominated the poor Oral Hygiene Index-Simplified scores group; this study concludes that stunting can affect the Oral Hygiene Index-Simplified scores

## Data Availability

Not applicable.
